# Disruption of neonatal Purkinje cell function underlies injury-related learning deficits

**DOI:** 10.1073/pnas.2017876118

**Published:** 2021-03-09

**Authors:** Aaron Sathyanesan, Panagiotis Kratimenos, Vittorio Gallo

**Affiliations:** ^a^Center for Neuroscience Research, Children’s National Research Institute, Children’s National Hospital, Washington, DC, 20010;; ^b^Department of Pediatrics, Division of Neonatology, Children’s National Hospital, Washington, DC, 20010;; ^c^The George Washington University School of Medicine and Health Sciences, Washington, DC, 20037

**Keywords:** cerebellum, neonatal brain injury, Purkinje cell, adaptive behavior, locomotor learning

## Abstract

It is hypothesized that perinatal cerebellar injury leads to long-term functional deficits due to circuit dysmaturation. Using a novel integration of GCaMP6f fiber photometry with automated measurement of cerebellar behavior using the ErasmusLadder, we causally link cerebellar injury to altered Purkinje cell responses during maladaptive behavior. Chemogenetic inhibition of neonatal Purkinje cells is sufficient to phenocopy the effects of perinatal cerebellar injury. Our results uncover a direct link between perinatal cerebellar injury and activity-dependent maturation of cerebellar cortex.

## Significance

Injury to the cerebellum during late fetal and early postnatal life is associated with long-term motor and cognitive deficits. It is thought that injury at this stage of development results in delayed maturation of neural circuitry, causing altered behavior at later stages. This study identifies the neural basis of locomotor learning deficits in the cerebellum using a clinically relevant model of neonatal brain injury. By combining fiber-optic-enabled Purkinje cell activity measurement during locomotor behavior, we provide evidence for long-term changes in neuronal responses during learning. By artificially reducing Purkinje cell function during the neonatal stage, we observed similarly altered physiological responses as those seen in injury. Our findings indicate that injury-related inhibition of developing Purkinje cells causes long-term locomotor dysfunction.

Perinatal complications of preterm or term neonates often result in adaptive behavioral deficits. While injury to the developing cerebellum has been correlated to long-term behavioral abnormalities, especially in locomotor function, the specific neural circuits and physiological mechanisms that are disrupted are unknown. Recent work characterized the spatial and temporal components of interlimb coordination in cerebellum-dependent locomotor learning (1). However, the sparsity of techniques available to directly measure neuronal activity during adaptive cerebellar behavior hampers efforts to identify the functional and mechanistic basis of behavioral pathology.

We designed a unique method to measure Purkinje cell (PC) activity during an adaptive cerebellum-dependent locomotor learning task. We utilized the ErasmusLadder––an automated behavioral system that can accurately quantify cerebellum-dependent locomotor learning and adaptive behavior. The ErasmusLadder enables the use of an associative conditioned-learning paradigm that is temporally tuned to define cerebellum-specific aspects of motor learning over multiple trials (2, 3). By directly integrating fiber photometry of the genetically encoded Ca^2+^ indicator—GCaMP6f—with the ErasmusLadder, we successfully and simultaneously recorded population responses of PCs in mice in real time during unrestrained behavior on the ErasmusLadder. We used our method to integrate PC activity measurement with adaptive behavioral quantification to identify a mechanistic basis for locomotor learning dysfunction in a clinically relevant mouse model of neonatal brain injury (4).

## Results

Cerebellar injury during the perinatal period is significantly associated with long-term neurodevelopmental deficits (5, 6). We had previously defined specific long-term locomotor-learning abnormalities in the chronic perinatal hypoxia model of neonatal brain injury (2). However, in order to directly identify the cerebellar basis of locomotor-learning deficits, it is necessary to measure PC activity during locomotor behavior and adaptive learning. Specifically, in the context of the ErasmusLadder paradigm, learning is indicated by an anticipatory adjustment of step dynamics in response to a high-pitched tone (conditioning stimulus, CS) in order to avoid a randomly presented obstacle (unconditioned stimulus, US) in the path of motion.

**Fig. 1.**
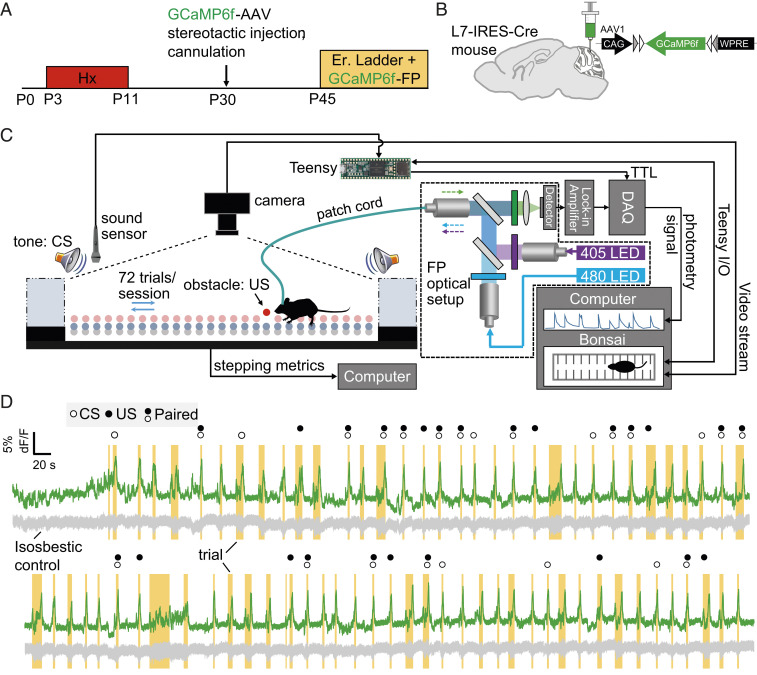
Experimental schematic and method design. (*A*) Experimental timeline for the chronic perinatal hypoxia model of neonatal brain injury followed by GCaMP6f-AAV injection and cannulation (P30). Mice are then used for ErasmusLadder experiments at P45. (*B*) Schematic showing injection site of Cre-dependent GCaMP6f AAV into cerebellar cortex. (*C*) Integration of GCaMP6f FP and ErasmusLadder behavioral system through Bonsai-enabled event detection and video time-locking. (*D*) Representative GCaMP6f dF/F data (green) from all 72 FP trials of an L7-IRES-cre mouse expressing GCaMP6f during a learning session. All trials (yellow) and CS events (open circle) are automatically marked using the sound sensor and video detection data received by the FP module through the Teensy-powered Bonsai platform. The gray trace represents the 410-nm isosbestic internal control signal which remains relatively unchanged across all trials. US, which is the obstacle randomly presented at some point during the trial, is denoted by a closed circle Trials with both CS and US marked are paired trials representing the associative conditioning task during the adaptive learning sessions.

How PC function changes during locomotor learning, specifically in the context of an associative learning paradigm, has not been completely characterized. It is also not known what the specific effect of neonatal brain injury is on PC function during locomotor learning. In order to determine how PCs respond during locomotor learning and the potential functional alterations caused by injury, we injected normal and injured L7-IRES-Cre animals (PC-specific Cre driver) with Cre-dependent GCaMP6f adeno-associated virus (AAV) into their cerebellar cortex, followed by implantation of a fiber-optic cannula (Fig. 1 *A* and *B*). Behavioral experiments were performed at P45. We interfaced the ErasmusLadder with GCaMP6f fiber photometry (FP) using the Bonsai visual programming framework and a Teensy board receiving event-sensor input (Fig. 1*C* and *SI Appendix*, Fig. S1). This enabled time-locking of animal movement and experiment-related events (such as high-pitched tone-conditioning stimulus or CS) to the FP signal from GCaMP6f-expressing PCs. Representative data from a GCaMP6f-injected normal L7-IRES-Cre mouse (Fig. 1*D*) indicated increased GCaMP6f dF/F (Fig. 1*D*, green trace) during the period that mice spent traversing the ladder (“trial,” Fig. 1*D*, all orange-shaded sections of recording), and relatively reduced dF/F when the mice were in the goal box (nonshaded). The GCaMP6f isosbestic internal control signal (410-nm excitation) was relatively stable (Fig. 1*D*, gray trace) across trials. Using our method, we could thus categorize and extract sections of the FP recording based on the different types of trials presented in a given learning session (Fig. 1*D*, “unperturbed,” unmarked; “CS only,” open circle; “US only,” solid circle; “paired,” open circle + solid circle).

**Fig. 2.**
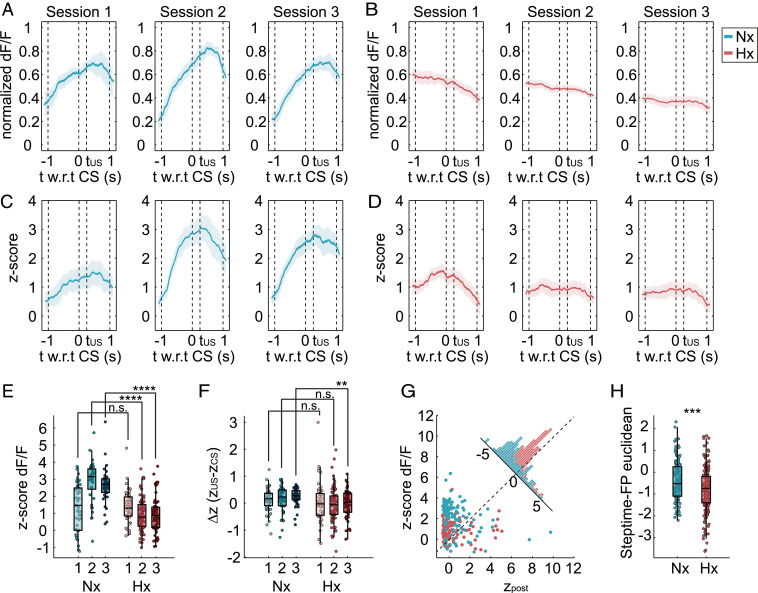
Purkinje cell dynamics are altered during adaptive cerebellar learning in Hx mice. (*A*) Representative GCaMP6f dF/F signal within a 2-s window with t = 0 as CS onset, from PCs in an Nx mouse, averaged across multiple learning trials from learning sessions 1,2, and 3. Blue line is mean; blue shading denotes confidence intervals. Dotted line at t = 0.25 denotes onset of US (t_US_), which is obstacle presentation. (*B*) Representative dF/F signals averaged over multiple trials in all learning sessions in a single Hx mouse (red). (*C*) z-scored GCaMP6f dF/F signal in Nx animals (averaged across a total of 126 learning trials from *n* = 3 mice) (*D*) z-scored GCaMP6f dF/F signal in Hx animals (averaged across a total of 185 learning trials from *n* = 3 mice). Shading represents confidence intervals. (*E*) z-score dF/F box and scatter-plot comparisons between groups across all learning trials. Mixed-model analysis results indicate overall statistical differences: session factor F (1.976, 183.7) = 7.076; *P* = 0.0011; z dF/F measurement factor F (1, 119) = 91.74; *P* < 0.0001. session × z dF/F factor F (2, 186) = 25.49; *P* < 0.0001. Asterisks denote results of multiple comparison tests using Sidak's test between groups in a particular session. ****P < 0.0001; n.s. not significant. (*F*) Delta-*z* (difference in z-scored dF/F between t = 0.25 and t = 0) comparison between groups indicates overall statistical differences in delta-*z* factor in mixed-model analysis: F (1, 119) = 6.984; *P* = 0.0093, but not in the session factor (F (1.945, 180.9) = 0.2021; *P* = 0.8112) or the session × Δ*z* factor [F (2, 186) = 0.5838; *P* = 0.5588]. Multiple comparisons test using Sidak's test showed statistical differences between groups in session 3 (*P* = 0.0095) but not in sessions 2 or 3. (*G*) Scatter plot of pooled z-score dF/F against z-scored postperturbation step time from all sessions. Corner histogram shows projected distributions indicating distinct profiles for Nx and Hx. Note that Nx group has higher z-score dF/F for similar or lower z postperturbation step time, compared to Hx. (*H*) Log-transformed step-time–FP Euclidean distance measurement (incorporating z-scored steptimes and z-score dF/F) comparison between Nx and Hx groups. Welch's *t* test analysis: *P* = 0.0004, t = 3.565, df = 248.1, mean of Nx = -0.3850, mean of Hx = -0.7993. F test to compare variance: F, DFn, Dfd: 1.091, 180, 113; *P* = 0.6174.

To identify potential differences in learning-correlated PC function between mice raised in normoxic conditions (blue, Nx) and mice with cerebellar injury (red, Hx), we compared FP signals specifically from paired trials, during which the CS is paired with the US with an interstimulus interval (250 ms). Representative FP signals (blue trace) from an Nx animal across multiple trials showed a rapid increase in the CS–US window during session 1 of learning (Fig. 2*A*, session 1). This increase was also observed in sessions 2–3 (Fig. 2*A*). FP signals obtained from an Hx animal showed a slight increase in session 1, but remained relatively unchanged in remaining learning sessions, indicative of PC dysfunction during adaptive, associative, cerebellum-dependent learning (Fig. 2*B*). z-scored dF/F signals across multiple trials and animals showed a similar pattern in the data. Mixed effects analysis indicated significant differences in mean z-score dF/F between Nx (Fig. 2*C*) and Hx (Fig. 2*D*) groups (*P* < 0.0001; Nx: total 126 trials from *n* = 3 mice; Hx: total 185 trials from *n* = 3 mice). Multiple comparison tests between groups for a particular session showed no statistically significant difference between z-Nx and Hx z-scores, indicative of relatively similar levels of PC activity. However, for learning sessions 2 and 3, significant differences were seen, indicating higher PC activity in the CS-US window (Fig. 2*E*). Comparing z-score differences between US onset and CS onset (Fig. 2*F*), we found overall significant statistical difference in the Δ*z* factor (*P* = 0.0093), but not in the session factor (*P* = 0.8112) or the session × Δ*z* interaction factor (*P* = 0.5588). To further identify the link between dysfunctional PC activity and behavior, we analyzed the distribution of z-score dF/F in the CS–US window with respect to postperturbation step-time measurements during the time window within the same trial (Fig. 2*G*). We found that, across multiple trials and animals, z-score dF/F and z-scored postperturbations are distributed distinctly between groups, with little to some overlap (Fig. 2*G*, corner histogram). Nx animals had higher z-score dF/F and lower z-scored postperturbation step times. In contrast, Hx animals had lower z-score dF/F for similar or higher z-score postperturbation step time. This strongly indicates that increased adaptive learning tracks well with increased PC activity during the CS–US window. Further, analysis of Euclidean distance between [z-scored preperturbation, z-score dF/F pre-CS] and [z-scored postperturbation, z-score dF/F CS-US] revealed a higher Euclidean distance for Nx compared to Hx (*P* = 0.0004), indicating that the difference in PC activity corresponding to preperturbation and postperturbation step times was more pronounced for Nx than Hx (Fig. 2*H*), reflective of higher PC efficiency in Nx mice. Together, this provides direct evidence that cerebellar behavioral deficits in neonatal brain injury are due to dysfunctional PC responses when challenged to adapt during learning.

Are PCs in Hx animals generally nonresponsive to any kind of stimuli? To identify the extent and context of attenuated responsiveness in PCs of mice with cerebellar injury, we analyzed FP signals from PCs during the pretrial air puff––which is a cue for mice to leave the goal box and enter the ladder behavioral track (*SI Appendix*, Fig. S2*A*). Both Nx (*SI Appendix*, Fig. S2*B*) and Hx PCs (*SI Appendix*, Fig. S2*C*) were generally responsive to air stimulation, indicating that dysfunctional PC function during the CS–US window was specific to adaptive learning on the ErasmusLadder, and not due to a general lack of sensory responsiveness. Pooled averages of Nx and Hx PC responses in CS-only trials indicated a profile similar to that of paired trials in the respective groups (*SI Appendix*, Fig. S3*A*). Similar to the differences observed in paired trials, PC z-score dF/F in CS-only trials (between t = 0 and t = 0.25 s) was significantly higher in Nx compared to Hx (*P* < 0.0001; t = 7.606, dF = 76) (*SI Appendix*, Fig. S3*B*), thus offering further evidence of an associative cerebellum-dependent learning deficit in Hx animals. Interestingly, in US-only trials, although the pooled average profile of PC activity in Nx was somewhat different in temporal dynamics from Hx animals, both groups of PCs responded in a similar manner following US presentation––a rapid reduction in z-score dF/F (*SI Appendix*, Fig. S3 *C* and *D*), indicating that while there may be a potentially small difference in non–learning-related PC responses encoding locomotion, this difference may not significantly affect adaptive learning, since PC activity in response to a random obstacle without any preceding conditioning tone was not different between groups (*P* = 0.0716) (*SI Appendix*, Fig. S3*D*).

To identify behavioral outputs not directly related to adaptive learning that may also track with difference in PC activity during locomotor learning trials, we used generalized linear model regression. We did not observe obvious, consistent relationships across sessions between the different behavioral outputs and z-score dF/F or delta-*z* values (*SI Appendix*, Figs. S4 and S5 and Tables S1–S4). However, trial duration appeared to track modestly with *z* dF/F, with differences between Nx and Hx groups in that while z-score dF/F for Nx became invariant to trial duration by session 2; there was a tight positive linear correlation by the last learning session in the Hx group (*P* = 0.001; *SI Appendix*, Fig. S3*A*). Since velocity is known to be encoded in PC activity in nonhuman primate models (7), we also performed linear regressions of trial velocity with z-score dF/F and delta-*z* values (*SI Appendix*, Fig. S6 and Table S5). However, apart from a significant negative correlation between velocity and z-score dF/F in the Hx group (*P* < 0.01; session 3), we did not observe significant correlations in the data.

**Fig. 3.**
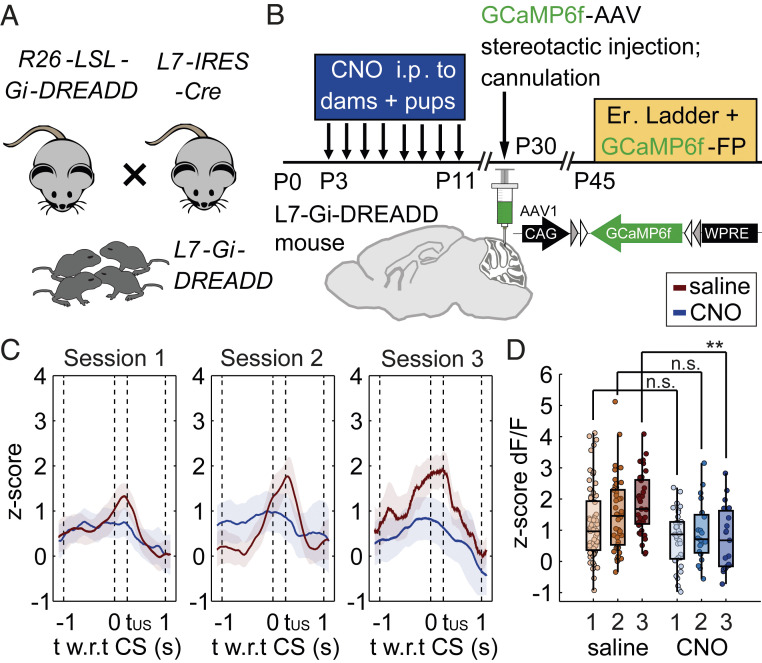
Purkinje cell-specific DREADD inhibition during P3-P11 phenocopies physiological impairments in Hx. (*A*) Inhibitory DREADD floxed (Gi-DREADD) mice were crossed to L7-IRES-Cre mice to obtain L7-Gi-DREADD offspring. (*B*) CNO was injected intraperitoneally (i.p.) to the dam twice a day between P3–P11 and to the pups between P7–P11. Stereotactic GCaMP6f injection into the cerebellar cortex and cannulation of L7-Gi-DREADD mice was performed at P30 and ladder experiments at P45 as before. (*C*) L7-GiDREADD mice injected with CNO during P3–P11 had altered Purkinje cell responses during learning trials (total of 74 trials from *n* = 3 mice) compared to L7-GiDREADD mice injected with saline (total of 139 trials from *n* = 4 mice), similar to that seen in Hx mice. Shading represents confidence intervals. (*D*) Purkinje cell z-score dF/F signal is low across learning sessions in CNO-treated control L7-Gi-DREADD mice and is significantly lower compared to PC activity z-scores for saline-injected L7-GiDREADD control mice. Mixed-effects analysis: session factor: F (1.975, 204.5) = 1.927, *P* = 0.1487; treatment factor: F (1, 207) = 20.75, *P* < 0.0001; session × treatment: F (2, 207) = 1.506, *P* = 0.2242; Sidak’s multiple comparison test: session 1: *P* = 0.0783; session 2: *P* = 0.1390; session 3: *P* = 0.0012.

Neuronal activity during early development is critical for proper circuit maturation, which if disrupted results in long-term synaptic and physiological deficits (8). During the early postnatal period, multiple activity-dependent processes occur in the developing cerebellar circuit, a majority of which directly involve PCs (9). We had previously shown that PC basal activity is drastically reduced in Hx animals at P13, suggesting that neonatal brain injury causes an early attenuation of PC function, which may potentially lead to long-term physiological and behavioral deficits. In order to define the precise connection between specific alterations in PC activity during development and behavioral abnormalities due to early cerebellar injury, we used an inhibitory chemogenetic approach using clozapine *N*-oxide (CNO) and designer receptors exclusively activated by designer drugs (DREADDs) to specifically attenuate PC activity during the same developmental time window as hypoxia-induced neonatal brain injury (Fig. 3 *A* and *B*). L7-Gi-DREADD mice were then injected with GCaMP6f AAV and cannulated. Fiber photometry and behavioral quantification was performed at P45. Similar to Hx animals, CNO-injected L7-Gi-DREADD animals have significantly increased postperturbation step times compared to saline-injected controls, indicative of a cerebellum-dependent adaptive learning deficit (*SI Appendix*, Fig. S7). CNO-mediated inhibition of Gi-DREADD–expressing PCs during development caused a long-term Hx-like response at P45, as evidenced by flattened GCaMP6f dF/F in the CS–US learning window compared to saline controls (saline-injected: total 139 learning trials from *n* = 4 mice; CNO-injected: total 74 learning trials from *n* = 3 mice) (Fig. 3*C*). Furthermore, chemogenetic inhibition of PCs during development also resulted in a lower PC activity z-scored dF/F during adaptive learning, which was most pronounced during the last session (Fig. 3*D*) [mixed effects analysis: treatment factor: *P* < 0.0001, F (1, 207) = 20.75; session factor: *P* = 0.1487, *F*(1.975, 204.5) = 1.927; session × treatment: *P* = 0.2242, *F*(2, 207) = 1.506; Sidak’s multiple comparison test: session 1: *P* = 0.0783; session 2: *P* = 0.1390; session 3: *P* = 0.0012], indicative of significant and cumulative PC dysfunction. The rate of change of PC activity indicated by slope of GCaMP6f signals across groups also showed significant differences between CNO-injected and saline-injected groups, similar to differences between Hx and Nx slopes (*SI Appendix*, Fig. S8). Within the context of locomotor function, it is unlikely that our results in these long-term experiments (behavior and physiology quantified more than 1 mo following the last CNO injection) are due to clozapine-mediated off-target effects since we did not observe any baseline change in general locomotor activity in the CNO-treated animals (preperturbation step-time comparison between saline-treated and CNO-treated: session 1: *P* = 0.9778; session 2: *P* = 0.9980; session 3: *P* = 0.9997). We also did not observe a significant difference in fraction of trials when animals left the goal box before the cue (saline-treated: 3.54%, and CNO-treated: 2.44% of total number of paired trials) versus those when animals left after the cue (saline-treated: 96.46%, and CNO-treated: 97.56% of total number of paired trials; Fisher’s exact test: *P* > 0.9999), suggestive of similar levels of activity prior to locomotion on the ladder, and similar propensity to leave the goal box.

The cerebellum is highly vulnerable to injury in the neonatal period resulting in significant long-term behavioral deficits (5, 10, 11). However, methods to shed light on causative cellular and physiological factors linking cerebellar injury to behavioral alterations at the level of circuitry have not been reported. Here we present a method combining fiber photometry and an adaptive cerebellar learning paradigm using the ErasmusLadder system to provide direct evidence of PC dysfunction associated with perinatal cerebellar injury during behavior. Using our method, we show that PCs show increased activity during ongoing locomotion and have a high average level of activity during learning and across subsequent sessions of adaptive locomotor learning. This level of PC activity is significantly attenuated in Hx animals. Next, we show that behavioral correlates of reduced PC activity in Nx mice are distinct from those in Hx mice. Finally, we show that specific inhibition of PCs using chemogenetics during a time window correlated to neonatal cerebellar injury produces the same physiological phenotype as seen in Hx animals during locomotor learning, indicating the pivotal role that PCs play in the development of adaptive cerebellar behavior.

Cerebellum-dependent learning has been mostly studied using very narrowly defined tasks, such as eyeblink conditioning or vestibular-ocular reflex adaptation learning. While these studies contributed to delineating cerebellar function and associated circuitry, it is relatively unclear to what extent cerebellar circuitry is involved in complex tasks such as whole-body locomotion and locomotor learning. Recently, locomotor adaptation learning using the split-belt paradigm has been used to elegantly dissociate the spatial and temporal components of locomotor adaptation in mice (1). However, locomotor adaptation using a split belt, while conserved in humans, is ethologically less relevant compared to adapting to an obstacle in the path of locomotion for both rodents and humans. Additionally, while previous studies have monitored PC activity during locomotion using electrophysiology or Ca^2+^ indicators (12–14), none have paired these methods with an adaptive learning or challenge paradigm. In this sense, our method of measuring PC activity during adaptive locomotor learning on the ErasmusLadder provides a better synthesis of the classical conditioning paradigm and a complex task, such as obstacle avoidance during active locomotion.

Perinatal disruption of development due to preterm birth and prematurity-induced brain injury is highly correlated to later behavioral abnormalities (15). The effect of hypoxic injury on the developing neonatal brain depends on the severity and duration of hypoxia, as well as on the developmental stage at which it occurs (16–18). Acute severe hypoxia in the full-term neonate (hypoxic-ischemic encephalopathy) results in cerebral cortex and basal ganglia injury, and less commonly in white-matter damage (19). In contrast, chronic hypoxia in the preterm neonate mainly affects the white matter and the cerebellum, and less often the cerebral cortex and basal ganglia, both in humans and mice (17, 20). We used an established mouse model of neonatal chronic Hx, which is clinically relevant to prematurity-induced brain injury in humans (16, 21). Similar to the brain injury observed in preterm neonates, this mouse model is characterized by atrophy of subcortical white matter, ventricular dilation, and cerebellar injury (21, 22). Of note, although neuronal loss is observed in the cerebral cortex concurrent with decreased cortical volume shortly after Hx, in late adulthood the total number of cortical neurons is not different from that of Nx mice (21, 23). In addition to these findings, diffusion tensor imaging showed no significant differences in the cerebral cortex between Hx and Nx (21). Most importantly, when older mice (P40–45) were assessed with tests of general activity and cognition, which reflect functionality of the cerebral cortex, there were no differences between Hx and Nx, further supporting the notion that the cerebral cortex is significantly regenerated in adulthood in this mouse model.

In contrast to these findings in cerebral cortex, we and others have previously shown that prematurity-induced cerebellar injury persists in adulthood––with a correlation between cerebellar injury and functional deficits persisting until later in life, both in humans and mice (2, 24, 25). In order to ensure that the locomotor learning deficits are predominately cerebellum-dependent, we morphologically analyzed another brain region––the posterior parietal cortex (PPC)––which may potentially contribute to controlling and learning the ability to step over an obstacle during locomotion (26) on the ErasmusLadder. Our histological analysis confirmed that neuronal cell count in the PPC as well as gross anatomical measurement of the entire cerebral cortex at P45 are not significantly different between Hx and Nx mice (*SI Appendix*, Fig. S9), providing additional evidence in support of the hypothesis that the cerebellum is a major locus of locomotor learning deficits (as assessed using the ErasmusLadder) in our mouse model of neonatal brain injury.

A slate of recent reports has identified cerebellar circuitry abnormalities in mouse models of neurodevelopmental disorders (27–29). Many of these reports implicate abnormal cerebellar development as being a key contributing factor leading to abnormal social behaviors. While noteworthy and novel, the role of early developmental disruption on cerebellar circuitry has not yet been fully explored with regards to the consequences on motor behavior––for which the cerebellum is generally deemed essential. In this context, our current work provides direct evidence for cerebellar injury-induced behavioral consequences due to early developmental disruption of locomotor cerebellar learning. Future studies using our method concurrently with optogenetic integration and higher resolution analysis of locomotor learning behavior using machine-learning approaches will enable further insights into the role of the developing cerebellum in physiologic and pathologic locomotor behavior.

## Materials and Methods

### Animals.

All animal procedures were performed according to the Institutional Animal Care and Use Committee of the Children’s National Health System (Protocol 30473) and the *Guide for the Care and Use of Laboratory Animals* (NIH) (30). We used both male and female sexes of different strains across all experimental groups.

### Chronic Perinatal Hypoxia.

Chronic perinatal hypoxia was used to induce neonatal brain injury. Briefly, *L7-IRES-Cre* (Jdhu strain; The Jackson laboratory) (31) or C57BL/6 pups were housed with surrogate CD1 dams from P3-P11 in a hypoxia chamber (Biospherix) at 10.5% FiO_2_, as previously described (2, 4). Mouse litters were randomly chosen to either undergo hypoxic rearing or serve as normoxic controls. At P11, mice were removed from the chamber and transferred to a room with normoxic air conditions. For ErasmusLadder experiments, Nx and Hx mice at P30 were used for AAV stereotaxic inject6on and cannula implantation. Mice were then used for ErasmusLadder experiments at P45. For histological experiments, mice were used at P45.

### Chemogenetics.

We used the Gi-DREADD strain (*R26-LSL-hM4Di-DREADD*; The Jackson Laboratory) (32) to chemogenetically silence Purkinje cells in a selective manner during development. Briefly, we crossed *Gi-DREADD-floxed* mice with *L7-IRES-Cre* animals to obtain animals in which Gi-DREADD was conditionally expressed in Purkinje cells. We injected the dam from P3-P11 with CNO (Tocris) at 10 mg/kg body weight twice per day, to increase bioavailability of CNO to pups via lactation. We injected pups with CNO at 5 mg/kg body weight once per day from P7-P11. Control mice were injected with the same volumes of saline as the CNO group.

### Surgical Procedures for Viral Injection and Cannulation.

We performed surgeries as described previously (2) with some modifications to include cannulation for fiber photometry experiments (33). Briefly, mice were induced and maintained in an anesthetic state using an Isoflurane system at 1–2%. Buprenorphine was injected into the scruff, followed by Lidocaine injection into the scalp. Then, the hair on the skull was trimmed using scissors and cleaned using Povidone Iodine solution. An incision was made to expose the skull above cortex, as well as the cerebellum. After cleaning of the fascia, 0.3% hydrogen peroxide was used to dry the skull. A hole was then drilled intracranially above the simple lobule, gently removing the dura with fine forceps. One to two injections of 1 µl GCamp6f AAV were made into the simple lobule at a depth of ∼0.75 μm. The simple lobule is known to be involved in cerebellar-dependent motor learning and memory (34, 35). We have previously targeted this region for in vivo electrophysiology experiments and observed deficits in PC firing in anesthetized animals (2) and therefore sought to further characterize PC activity specifically in this region of the cerebellum. Following injection, low autofluorescence mono–fiber-optic cannulas (0.48/0.66 NA; Doric lenses) were implanted at the same height and position sealed using dental cement. Recovery procedures were followed, including pain management, using Buprenorphine and antibiotics. Animals were left to recover from surgery for a period of 2 wk, and then used in experimental testing on the ErasmusLadder.

### Integration of ErasmusLadder and Fiber Photometry Setup.

We integrated the capabilities of the Erasmus Ladder to quantify cerebellar behavior in a locomotor context with a GCaMP6f fiber photometry system, using the visual programming platform *Bonsai* (36) and a Teensy board. First, we successfully developed a custom Bonsai workflow to detect and follow the mouse on the entire ladder through a camera videostream (*SI Appendix*, Fig. S1). We then used a Teensy 3.5 board that incorporated the detection of sound––using analog sound sensors (DF Robot)––to denote the onset of air cue in the goal box and the conditioning stimulus while the animal is on the ladder. We preferred the use of Teensy 3.5 (PJRC) due to its relatively fast processing speed and the ability to handle +5-V signals from sensors. Lastly, we used Bonsai to send TTL signals for video-enabled detection of mouse movement and sound sensor signals to the Tucker-Davis Technologies (TDT) Open bench fiber photometry module, in order to enable time-locking of air-cue onset and conditioning stimulus onset.

### Data Analysis Pipeline.

We performed all analysis on MATLAB (v. 2014 a) using custom routines and scripts. All TDT files with GCaMP6f signal data and external transistor-transistor logic (TTL) signals were converted and stored as .mat files using “TDT2mat.” Next, we removed artifacts from the data, followed by data processing, wherein we compared the 480-nm signal to the 410-nm internal control signal. Following background signal correction for photobleaching and normalization, we ensured that the dF/F signal at 480 nm showed robust differences compared to the 410-nm signal. To obtain z-scored plots, we first filtered the 410-nm signal using a Savitsky Golay filter in order to remove high-frequency noise. The 480-nm signal was then subtracted from the filtered 410-nm signal, background-adjusted, and then z-scored (37). We isolated the learning session trials for further analysis. From these trials, a 2-s window was isolated with respect to the CS onset as t = 0. This window was used to average z-score plots across trials obtained from different animals. We then measured mean z-scores within the CS–US window i.e., between t = 0 and t = 0.25 s. Mean z-scores within this window were used for hypothesis testing. We then measured Δz which we defined as difference in z-scores at t = 0.25 and t = 0. We also calculated z-scores for step-time data. Slopes were calculated for the data in the CS–US window that yielded the rate of change of GCaMP6f signal. Linear regressions were performed using the “polyfit” command. For step-time–FP–Euclidean measurements to combine behavioral and physiological readouts, we used the formula

$$\log_{2}\left(\sqrt{{\left(z_{post}-z_{pre}\right)}^{2}+{\left(z_{US}-z_{CS}\right)}^{2}}\right),$$

where $z_{post}$ and $z_{pre}$ are z-scored postperturbation and preperturbation step times respectively, and $z_{CS}$ and $z_{US}$ are GCaMP6f dF/F z-scores at onset of CS (t_CS_ = 0) and US (t_US_ = 0.25 s), respectively.

### Tissue Processing and Histology.

Histological analysis was performed at P45. Hypoxic or normoxic mice were anesthetized with isoflurane and transcardially perfused with 0.1 M phosphate buffered saline (PBS), pH 7.4, followed by 4% paraformaldehyde (PFA). Brains were postfixed in 4% PFA overnight, and then stored in PBS at 4 °C until further use. We utilized Golgi–Cox staining in conjunction with haematoxylin and eosin (H&E) staining to perform neuronal cell-counts in the posterior parietal cortex. Serial sagittal cryosections (60 μm) through the entire posterior parietal cortex were stained with FD Rapid GolgiStain kit by FD NeuroTechnologies, Inc.

### Image Acquisition and Analysis.

We used the PANNORAMIC MIDI II automatic digital slide scanner microscope from 3D Histech. We used the Point Gray GS3-U3-51S5M-C camera. Z-stack images of 1-μm-thick single planes were captured using the 3dhistech software version 1.23.1.71684. The camera adapter magnification was 0.63×, micrometer/pixel X: 0.273810, micrometer/pixel Y: 0.273810, output resolution: 36.521675. CaseViewer 2.4 edition software was used to acquire the images. Images were viewed using NIH ImageJ and the CaseViewer Image Browser. All histological quantifications were performed in a blinded manner. The PPC was identified according to the Allen Brain Atlas (mouse) (39) and Paxinos and Franklin's *The Mouse Brain in Stereotaxic Coordinates* (40). Neuronal cells (Golgi–Cox and H&E stain) were manually counted in each optical section using the ImageJ “Cell Counter” plugin at 40× per regions of interest with a perimeter of 1 mm. Data were obtained from five mice per group.

### Data Visualization and Statistics.

Data visualization and statistical plotting was performed on MATLAB (v. 2014a) using the Gramm data visualization toolbox (38) and Adobe Photoshop. Statistical analysis and hypothesis testing was conducted using GraphPad Prism. For quantitative comparisons, normality tests were performed and statistical comparison tests were selected appropriately. Outliers were removed based on Robust regression and outlier identification (ROUT analysis; Q = 1%). To compare mean z-scores within the CS–US window and Δ*z* we used mixed-effects model analysis with Geisser–Greenhouse correction. The Sidak test was used for correcting multiple comparisons between groups for a particular learning session. Multiplicity adjusted *P* values were obtained for comparison. To compare Euclidean measurements, we used the Mann–Whitney *u* test. Generalized linear model was used for regression analysis. To compare pooled z-score dF/F for CS-only trials, we used the unpaired *t* test. To compare pooled delta *z/z* in CS-only and US-only learning trials, and pooled z-score dF/F in US-only trials, we used the Mann–Whitney *u* test. To compare slopes between groups, we used the Kruskal–Wallis test with Dunn’s test to correct for multiple comparisons. To compare step times we used two-way repeated measures ANOVA (RM-ANOVA). Adjusted *P* values were reported for multiple comparisons. To compare neuronal cell counts and anatomical measurements we used the unpaired *t* test.

## Supplementary Material

Supplementary File

## Data Availability

Dataset data have been deposited in Zenodo (https://zenodo.org/) (41).
