# Supplementary material for: Disruption of neonatal Purkinje cell function underlies injury-related learning deficits
Source: Proc Natl Acad Sci U S A. 2021 Mar 9;118(11):e2017876118. doi: 10.1073/pnas.2017876118 (PMC7980280; doi:10.1073/pnas.2017876118)
Supplement: Supplementary File [file pnas.2017876118.sapp.pdf]

PNAS
www.pnas.org

Supplementary Information for

Disruption of neonatal Purkinje cell function underlies injury-related learning deficits

Aaron Sathyanesan, Panagiotis Kratimenos, and Vittorio Gallo

Co-corresponding authorship: Vittorio Gallo, Aaron Sathyanesan
Lead contact: Vittorio Gallo
Email: vgallo@childrensnational.org (Vittorio Gallo)
asathyanesan@childrensnational.org (Aaron Sathyanesan)

This PDF file includes:

Supplementary Figures 1 to 9

Supplementary Tables 1 to 6

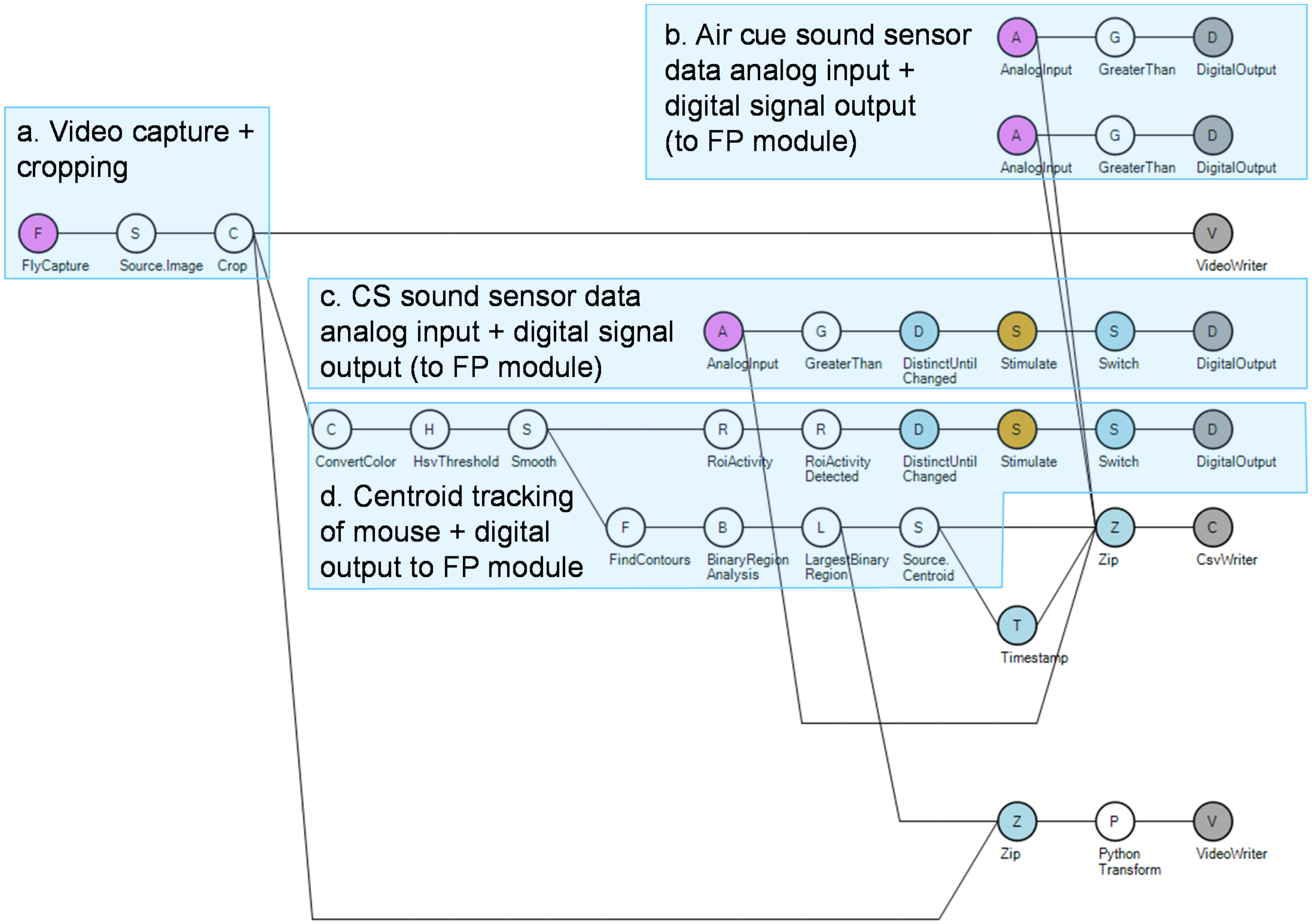

Supplementary Figure 1. a. Video capture is accomplished using the flycapture node in Bonsai for the blackfly USB3.0 camera and cropped to extract only the region of interest – the ladder track **b.** Analog sound sensors placed in each goal box provides the air cue onset information, which is the relayed to Bonsai, thresholded, and converted to digital output **c.** Conditioning stimulus (CS) onset is determined based on data from a

dedicated sound sensor, thresholded and converted to digital output to FP module **d.** Centroid tracking algorithm to capture movement on the ladder, which is then converted to digital signal output to FP module.

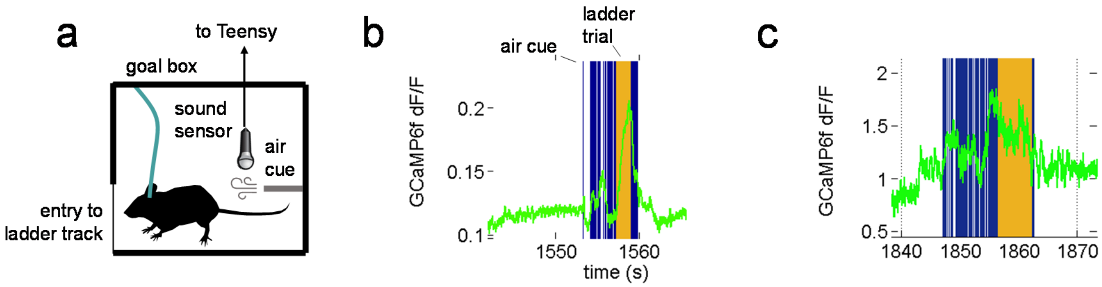

Supplementary Figure 2. Purkinje cells are generally responsive to sensory stimulation in Hx mice. a. The ErasmusLadder delivers an air cue in order for mice to leave the goal box to enter onto the ladder track. Air cue onset is determined via sound sensor data which is then relayed to Bonsai via the Teensy board **b.** The beginning of the ErasmusLadder trial showing the GCaMP6f dF/F signal (green) in Nx mice and increase in signal during the air cue (blue) **c.** GCaMP6f dF/F signal in Hx mice also show a rapid increase at the onset of air cue (blue). Ladder trials are denoted by the yellow bars.

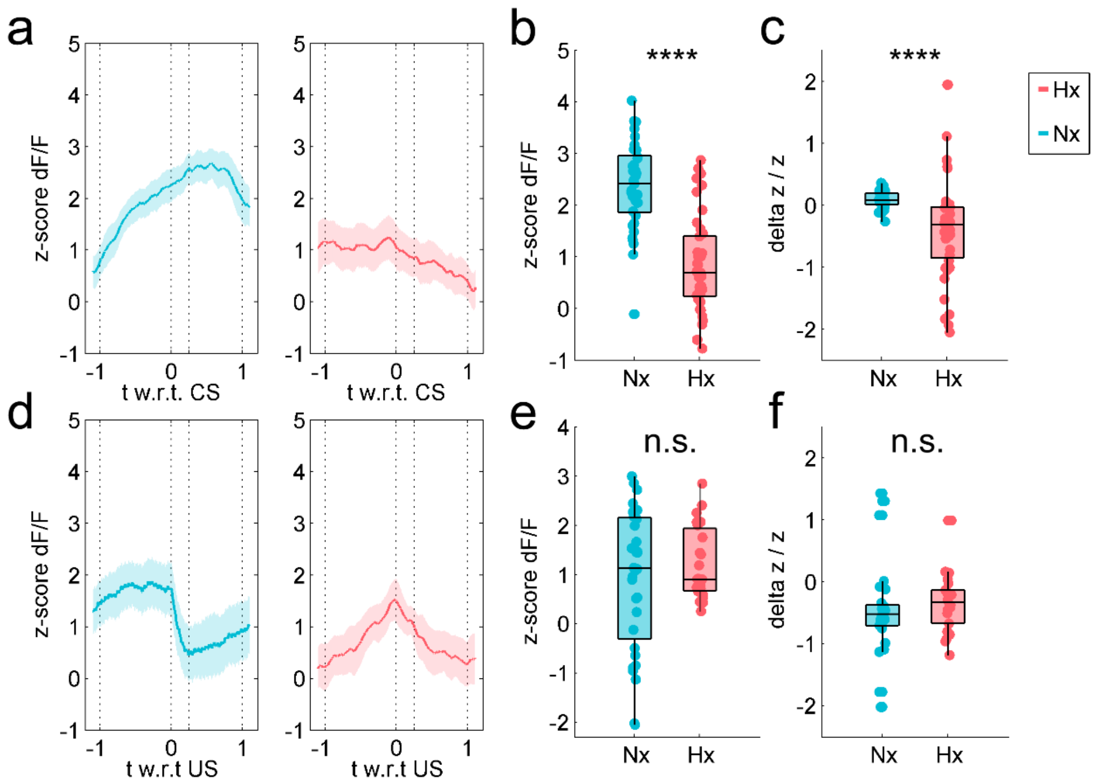

Supplementary Figure 3. Purkinje cells responses in CS-only and US-only trials. a. Pooled average PC responses in CS-only trials in Nx (blue; averaged across three sessions with a total of 37 CS-only trials from n = 3 mice) and Hx (red; averaged across three sessions with a total of 42 CS-only trials from n = 3 mice). **b.** Comparison of z-score dF/F (between t = 0 and t = 0.25) during CS only trials between Nx and Hx. Unpaired t-test (two-tailed): $P < 0.0001$; $t = 7.606$, $dF = 76$. **c.** Comparison of delta z / z in CS-only trials between groups. Delta z / z represents change in z score 250 ms after CS (t = 0.25), relative to z-score at t = 0. Mann-Whitney test: $P < 0.0001$ (exact); sum of ranks in Nx: 1591, Hx: 1037; Mann-Whitney U: 217; Difference between medians: Actual = -0.3874, Hodges-Lehmann = -0.4445. **d.** Pooled average PC responses in US-only trials in Nx (blue; averaged across three sessions with a total of 32 US-only trials from n = 3 mice) and Hx (red; averaged across three sessions with a total of 23 US-only trials from n = 3 mice). **e.** Comparison of z-score dF/F (between t = 0 and t = 0.25, w.r.t US onset) during US only trials between Nx and Hx. Mann-Whitney test: $P = 0.0716$ (exact), sum of ranks in Nx: 538, Hx: 1002; Mann-Whitney U: 262; Difference between medians: Actual = 0.2076; Hodges-Lehmann = 0.6809. **f.** Comparison of delta z / z in US-only trials between groups. Mann-Whitney test: $P = 0.2029$ (exact), sum of ranks in Nx: 420, Hx: 855; Mann-Whitney U: 262; Difference between medians: Actual = 0.2076; Hodges-Lehmann = 0.6809.

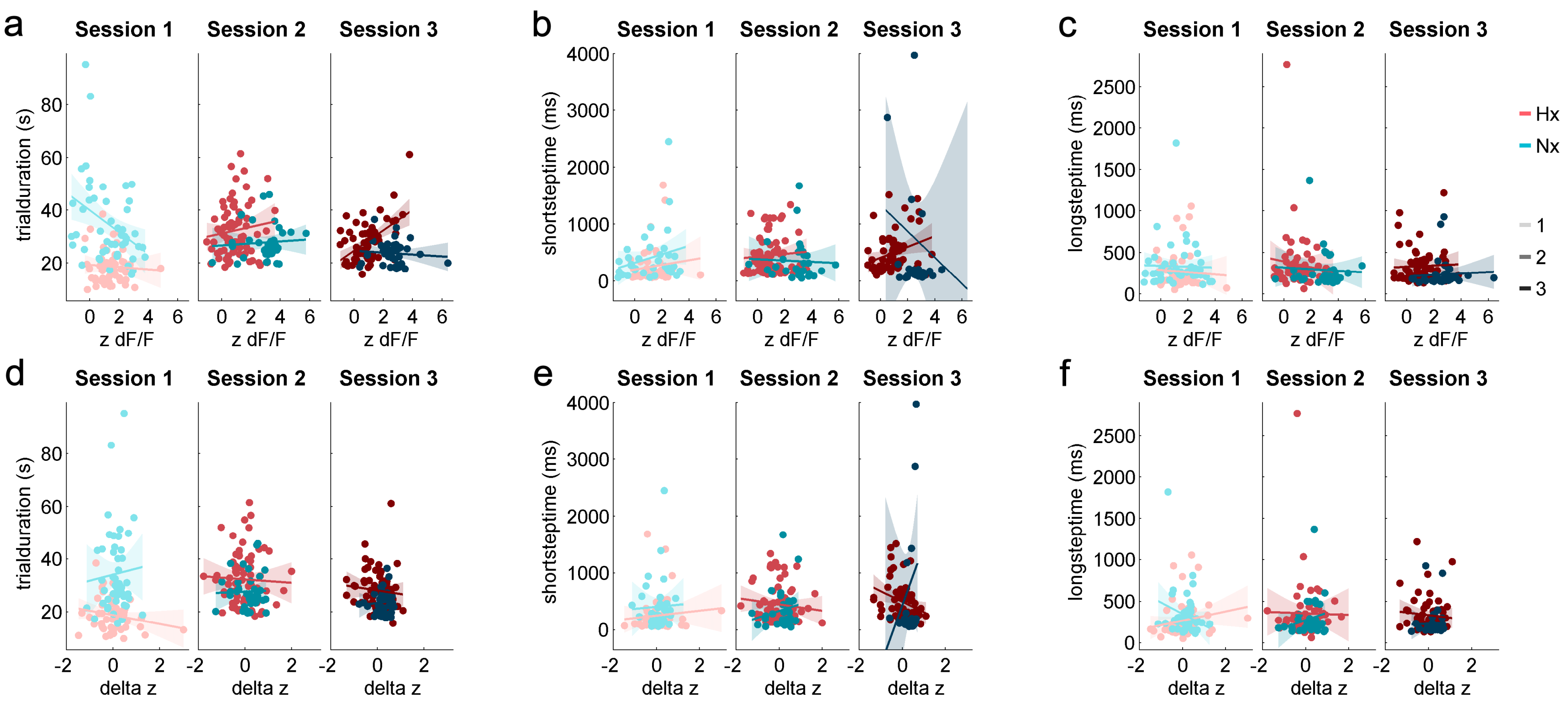

Supplementary Figure 4. Correlation between PC activity during CS-US learning window and other per-trial behavioral measurements obtained during learning trials. Linear regressions across learning sessions 1, 2, and 3, between z-score dF/F and **a.** total trial duration (goal-box to goal-box) **b.** average per-trial steptime of short-steps ($step_n \rightarrow step_{n+1}$ on one-side) and **c.** average steptime per trial of long-steps ($step_n \rightarrow step_{n+2}$ on one-side). Linear regressions across learning sessions 1, 2, and 3, between delta z-score (within t = CS onset and t = US onset) dF/F and **d.** total trial duration **e.** average steptime per trial of short-steps and **f.** average steptime per trial of long-steps. Regression equations and P-values are detailed in supplementary tables 1 and 2.

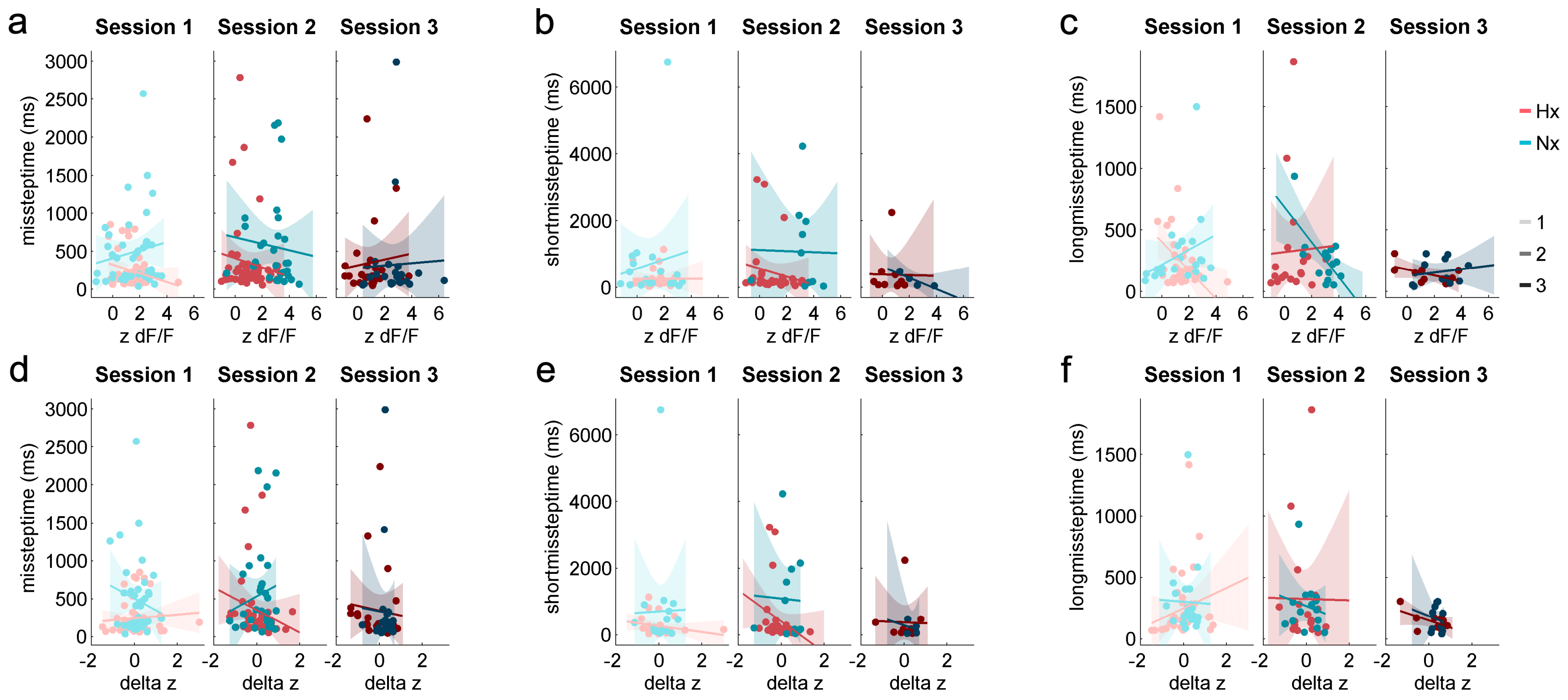

Supplementary Figure 5. Correlation between PC activity during CS-US learning window and per-trial miscoordination-related behavioral measurements obtained during learning trials. Linear regressions across learning sessions 1, 2, and 3, between z-score dF/F and **a.** per-trial average steptimes of missteps **b.** average per-trial steptimes of short missteps ($step_n \rightarrow misstep_{n+1}$) **c.** average per-trial steptime of long missteps ($step_n \rightarrow misstep_{n+2}$). Linear regressions across learning sessions 1, 2, and 3, between delta z-score (within t = CS onset and t = US onset) dF/F and **d.** total trial duration **e.** average per-trial steptimes of short-missteps **f.** average per-trial steptimes of long-missteps. Regression equations and P-values are detailed in supplementary tables 3 and 4.

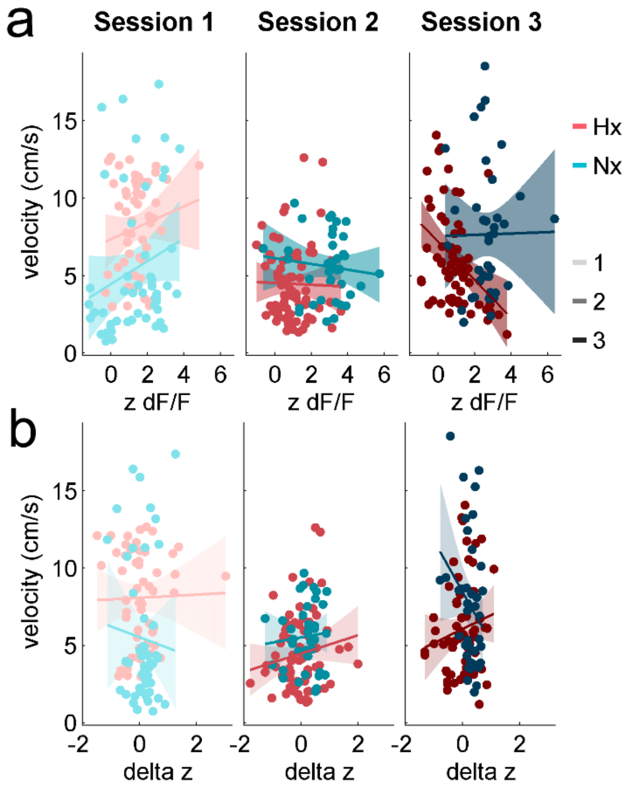

Supplementary Figure 6. Correlation between PC activity during CS-US learning window and velocity in learning trials. Linear regressions across learning sessions 1, 2, and 3, between velocity and **a.** z-score dF/F and between velocity and **b.** delta z-score. Linear regression equations and fitting values are detailed in supplementary table 5.

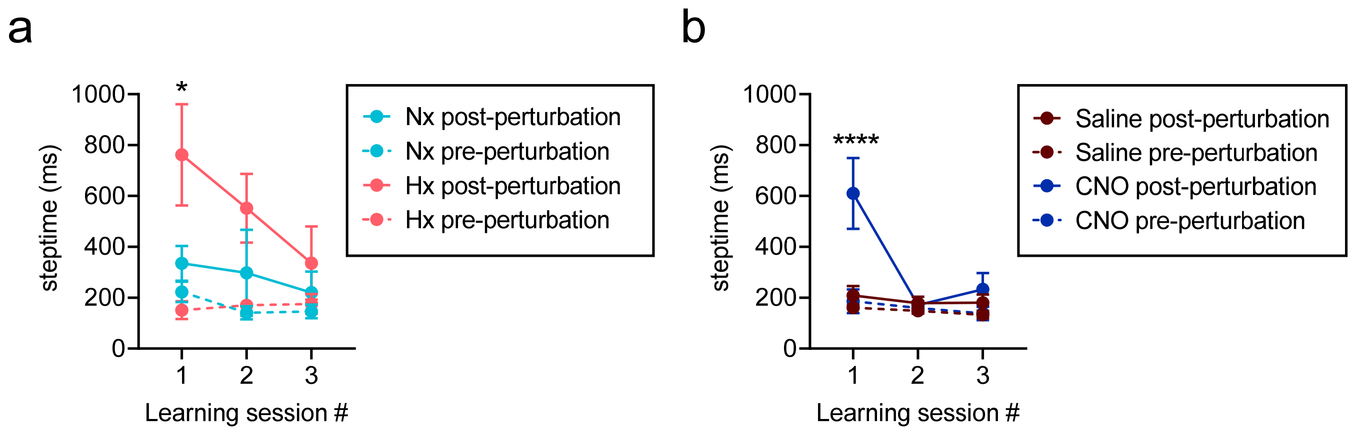

Supplementary Figure 7. Adaptive cerebellar-dependent learning measured using the ErasmusLadder. a. Comparison of post-perturbation steptimes in paired learning trials between P45 Nx (blue; n = 3 mice) and Hx (red, n = 3 mice) during fiber photometry measurement of PC activity. Two-way RM-ANOVA: Overall - treatment factor: $F (3, 8) = 6.508$ ($P = 0.0154$), session factor: $F (6, 16) = 1.199$ ($P = 0.3560$); session × treatment factor: $F (6, 16) = 1.199$ ($P = 0.3560$); Tukey's multiple comparison: Nx post-perturbation vs. Hx post-perturbation: session 1: Adjusted $P = 0.0321$, session 2: Adjusted $P = 0.3165$, session 3: Adjusted $P = 0.8497$ **b.** Comparison of post-perturbation steptimes in paired learning trials between P45 saline-injected (brown; n = 4 mice) and CNO-injected (navy; n = 3 mice) L7-Gi-DREADD mice during fiber photometry measurement of PC activity. Saline or CNO was injected to dams and pups between P3-P11 (pup age). Two-way RM-ANOVA: Overall - treatment factor: $F (3, 10) = 6.929$ ($P = 0.0084$), session factor: $F (2, 20) = 14.76$ ($P = 0.0001$); session × treatment factor: $F (6, 20) = 8.179$ ($P = 0.0001$); Tukey's multiple comparison: Saline-injected post-perturbation vs. CNO-injected post-perturbation: session 1: Adjusted $P < 0.0001$, session 2: Adjusted $P = 0.9995$, session 3: Adjusted $P = 0.8377$.

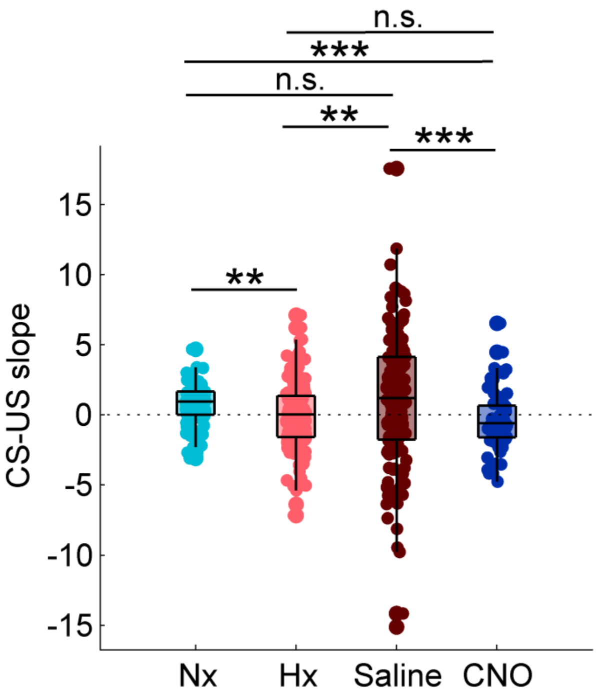

Supplementary Figure 8. Comparison of slopes of z-scored GCaMP6f signal in the CS-US window. Slope of z-scored GCaMP6f signal shows differences between groups in that that Nx and developmental saline-injected L7-Gi-DREADD groups have significantly higher median slopes compared to Hx and developmental CNO-injected L7-Gi-DREADD groups respectively. Note that slopes for Nx and saline groups are not significantly different. Similarly, Hx and CNO-injected groups are not significantly different. Kruskal-Wallis test: Overall P < 0.0001; Kruskal-Wallis statistic = 28.50; Nx vs. Hx: P = 0.0014; Nx vs. saline: P > 0.9999; Nx vs. CNO: P = 0.0008; Hx vs. saline: P = 0.0013; Hx vs. CNO: P > 0.9999; saline vs. CNO: P = 0.0008.

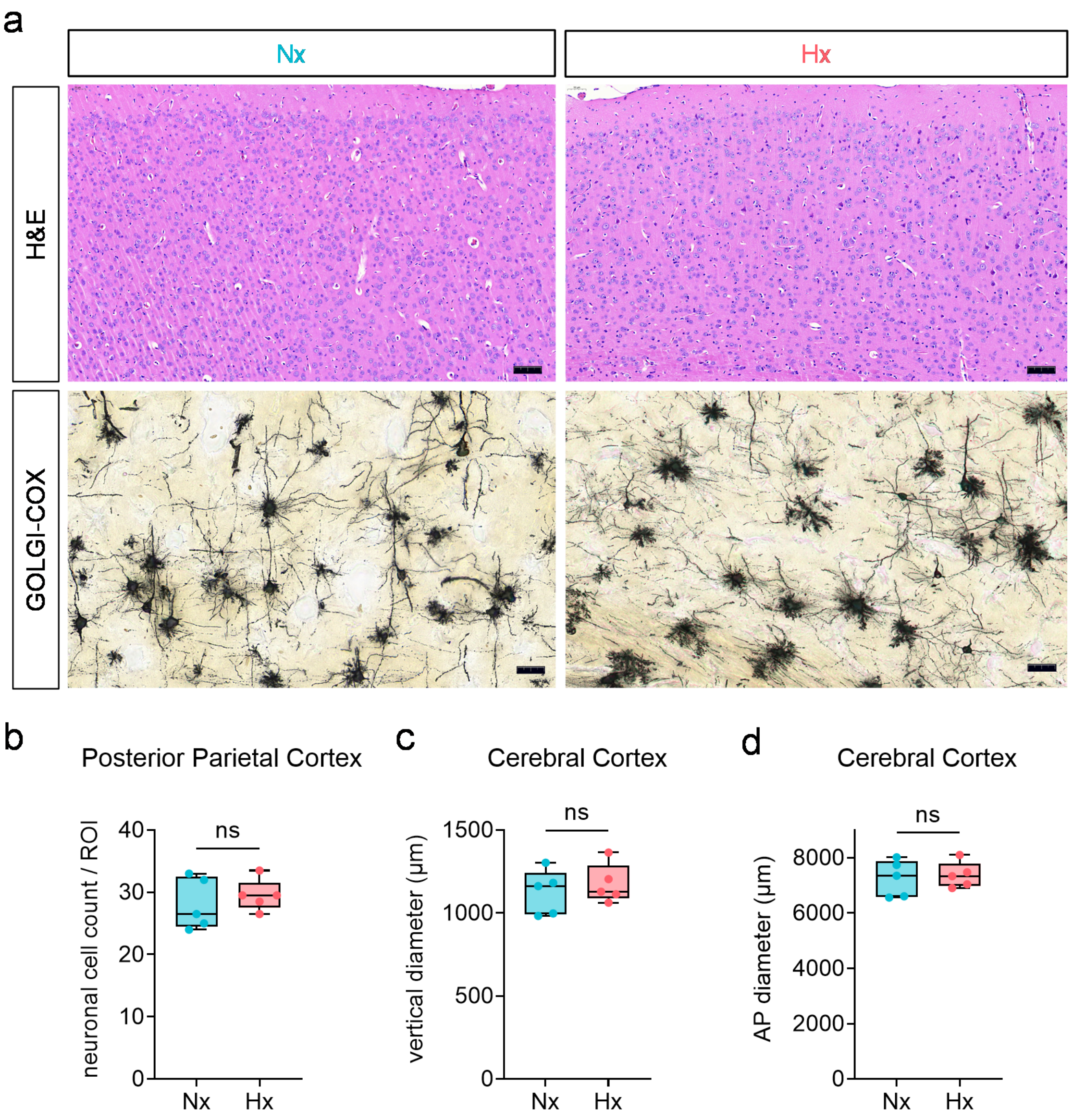

Supplementary Figure 9. Effect of Hx on the posterior parietal cortex. a. Representative photomicrographs of hematoxylin and eosin (H&E) and Golgi-Cox staining of posterior parietal cortex (PPC) at P45 in normoxia (Nx) and hypoxia (Hx). **b.** The number of neurons of the PPC was not different between Hx and Nx mice. N = 5 mice/group, Unpaired t-test: P = 0.5369, t=0.6451, df=8. **c.** In P45 mice, there was no difference in vertical-sagittal cerebral cortical diameter (N = 5 mice per group, Unpaired t-test: P = 0.7564, t=0.3210, df=8) and **d.** antero-posterior cerebral cortical diameter between Hx and Nx mice (N = 5 mice per group, Unpaired t-test: P = 0.7564, t=0.3210, df=8). In panel ‘a’, scale bar is 50 µm in the upper sub-panels (H&E) and 10 µm in the lower sub-panels (Golgi-Cox). Neuronal cells (Golgi-Cox and H&E stain) were manually counted in each optical section at 40X magnification per regions of interest (ROI) with a perimeter of 1mm.

Supplementary Table 1. Linear regression equations and P-values for intercept and slope (* P < 0.05, ** P < 0.01, *** P < 0.001, **** P < 0.0001) linked to Supplementary figure 4 (a-c). Columns are color-coded to match sessions and groups.

	Session 1		Session 2		Session 3	
	Nx	Hx	Nx	Hx	Nx	Hx
Trial duration vs. z dF/F	39.946*** - 4.1582** x	19.276*** - 0.43152x	26.638*** + 0.3831 x	31.163*** + 1.2459 x	24.624*** - 0.36077 x	24.665*** + 3.866*** x
Shortsteptime vs. z dF/F	321.68** + 74.929 x	203.14* + 41.191 x	384.69* - 11.475 x	430.82*** + 23.674 x	1331.7 - 231.07 x	412.03*** + 95.115* x
Longsteptime vs. z dF/F	339.31*** - 5.4873 x	289.19*** - 13.654 x	318.48** - 9.9532 x	391.36*** - 35.184 x	212.74** + 8.4116 x	327.61*** + 7.6649 x

Supplementary Table 2. Linear regression equations and P-values for intercept and slope (* P < 0.05, ** P < 0.01, *** P < 0.001, **** P < 0.0001) linked to Supplementary figure 4 (d-f). Columns are color-coded to match sessions and groups.

	Session 1		Session 2		Session 3	
	Nx	Hx	Nx	Hx	Nx	Hx
Trial duration vs. delta z	34.132*** + 2.298 x	18.728*** - 1.6834 x	27.689*** + 0.40463 x	32.287*** - 0.6499 x	23.412*** + 1.1441 x	28.107*** - 1.3681 x
Shortsteptime vs. delta z	409.69*** + 35.668 x	254.96*** + 43.189 x	338.19*** + 122.55 x	451.55*** - 56.388 x	448.98 + 1050.8 x	495.12*** - 185.63* x
Longsteptime vs. delta z	347.9*** - 137.26 x	269.08*** + 55.39 x	275.67*** + 90.707 x	356.59*** - 9.7341 x	231.99*** + 12.177 x	334.3*** - 31.623 x

Supplementary Table 3. Linear regression equations and P-values for intercept and slope (* P < 0.05, ** P < 0.01, *** P < 0.001, **** P < 0.0001) linked to Supplementary figure 5 (a-c). Columns are color-coded to match sessions and groups.

	Session 1		Session 2		Session 3	
	Nx	Hx	Nx	Hx	Nx	Hx
Missteptime vs. delta z	502.18*** - 153.91 x	243.98*** + 25.065 x	536.61*** + 156.66 x	350.13*** - 149.3 x	337.35 - 55.217 x	344.61** - 64.606 x
Short-missteptime vs. delta z	711.49 + 42.607 x	269.28*** - 90.43 x	1093.1 - 71.794 x	405.56** - 467.56 x	290.08 - 220.28 x	381.94 - 28.195 x
Long-missteptime vs. delta z	304.06*** - 13.971 x	252.57*** + 82.777 x	267.45*** - 69.67 x	326.56* - 5.4292 x	186.16* - 70.06 x	150.68*** - 58.377 x

Supplementary Table 4. Linear regression equations and P-values for intercept and slope (* P < 0.05, ** P < 0.01, *** P < 0.001, **** P < 0.0001) linked to Supplementary figure 5 (d-f). Columns are color-coded to match sessions and groups.

	Session 1		Session 2		Session 3	
	Nx	Hx	Nx	Hx	Nx	Hx
Missteptime vs. z dF/F	407.99** + 53.194 x	321.31*** - 57.647 x	681.86* - 42.615 x	407.08*** - 61.127 x	280.84 + 15.511 x	312.39* + 39.435 x
Short-missteptime vs. z dF/F	567.26 + 136.61 x	256.75* + 0.83652 x	1119.7 - 16.24 x	563.68** - 122.16 x	615.85 - 167.2 x	388.13 - 10.99 x
Long-missteptime vs. z dF/F	217.27* + 60.967 x	400.56*** - 115.65* x	679.53*** - 141.83** x	319.47* + 13.871 x	125.94 + 13.686 x	177.08*** - 23.763 x

Supplementary Table 5. Linear regression equations and P-values for intercept and slope (* P < 0.05, ** P < 0.01, *** P < 0.001, **** P < 0.0001) linked to Supplementary figure 6 (a, b). Columns are color-coded to match sessions and groups.

	Session 1		Session 2		Session 3	
	Nx	Hx	Nx	Hx	Nx	Hx
Velocity vs. z dF/F	4.4707*** + 0.73477 x	7.3579*** + 0.5285 x	6.1235*** - 0.18686 x	4.5278*** - 0.058257 x	7.5356*** + 0.044403 x	7.1617*** - 1.2274** x
Velocity vs. z dF/F	5.5316*** - 0.69593 x	8.0791*** + 0.10384 x	5.5343*** + 0.3381 x	5.5343*** + 0.3381 x	8.4164*** -3.2939 x	6.0809*** + 0.89654 x

Supplementary Table 6. Difference from zero measured for z-score dF/F for Nx and Hx groups for all sessions using a one-sample t test.

Group / Session	Nx - session 1	Nx - session 2	Nx - session 3	Hx - session 1	Hx - session 2	Hx - session 3
Theoretical mean	0.000	0.000	0.000	0.000	0.000	0.000
Actual mean	1.334	2.894	2.625	1.370	0.9208	0.8994
Number of values	50	37	39	53	71	61
t, df	t=6.748, df=49	t=14.37, df=36	t=15.92, df=38	t=10.80, df=52	t=8.014, df=70	t=6.848, df=60
P value (two tailed)	<0.0001	<0.0001	<0.0001	<0.0001	<0.0001	<0.0001
P value summary	****	****	****	****	****	****
Significant (alpha=0.05)?	Yes	Yes	Yes	Yes	Yes	Yes
